# Discovery of Novel Proteasome Inhibitors Using a High-Content Cell-Based Screening System

**DOI:** 10.1371/journal.pone.0008503

**Published:** 2009-12-30

**Authors:** Irena Lavelin, Avital Beer, Zvi Kam, Varda Rotter, Moshe Oren, Ami Navon, Benjamin Geiger

**Affiliations:** 1 Department of Molecular Cell Biology, Weizmann Institute of Science, Rehovot, Israel; 2 Department of Biological Regulation, Weizmann Institute of Science, Rehovot, Israel; University of Delhi, India

## Abstract

The regulated degradation of damaged or misfolded proteins, as well as down-regulation of key signaling proteins, within eukaryotic and bacterial cells is catalyzed primarily by large, ATP-dependent multimeric proteolytic complexes, termed proteasomes. Inhibition of proteasomal activity affects a wide variety of physiological and pathological processes, and was found to be particularly effective for cancer therapy. We report here on the development of a novel high throughput assay for proteasome inhibition using a unique, highly sensitive live-cell screening, based on the cytoplasm-to-nucleus translocation of a fluorescent proteasome inhibition reporter (PIR) protein, consisting of nuclear localization signal-deficient p53 derivative. We further show here that mdm2, a key negative regulator of p53 plays a key role in the accumulation of PIR in the nucleus upon proteasome inhibition. Using this assay, we have screened the NCI Diversity Set library, containing 1,992 low molecular weight synthetic compounds, and identified four proteasome inhibitors. The special features of the current screen, compared to those of other approaches are discussed.

## Introduction

The proteasome is the major proteolytic complex, responsible, in eukaryotic cells, for the degradation of a multitude of cellular proteins. This multi-protein complex, present in both the cytoplasm and the nucleus, catalyzes the ATP-dependent proteolysis of short-lived regulatory proteins, as well as the rapid elimination of damaged and abnormal proteins [1], [2]. The 26S proteasome is a large complex of ∼2.5 MDa. Based on biochemical analyses, this complex can be dissociated into two functionally distinct subcomplexes, the 20S core particle (CP) which is the proteolytic component, and the 19S regulatory particle (RP), that is responsible for recognizing, unfolding, and translocating polyubiquitinated substrates into the 20S CP, where they are degraded.

The 20S CP is a 670 kDa barrel-shaped protein complex made up of four stacked, seven-membered rings (4×7 subunits), two outer α rings and two inner β rings (α_1-7_β_1-7_β_1-7_α_1-7_). The two matching α rings are situated in the outer rims of the barrel, facing the 19S regulatory complex. The proteolytic active sites are located on the two identical β-rings, which are positioned in the center of the 20S complex [3], [4]. In eukaryotes, the catalytic activities of the proteasomes are confined to only three of the β-subunits. Although proteasomes can hydrolyze the amide bonds between most amino acids, proteolytic activities measured using fluorogenic substrates define three distinct (although not conclusive) cleavage preferences [5]: β2 possesses tryptic activity (i.e., cleaving after basic residues); β5 displays chymotryptic activity (i.e., cleaving after hydrophobic residues); and β1 has “caspase-like” or “post-acidic” activity. In all three active β-subunits, proteolytic activity is associated with their N-terminal threonine residue, which acts as a nucleophile in peptide-bond hydrolysis.

The use of proteasome inhibitors as drug candidates emerged from the observation that at specific concentrations, they can induce apoptosis in certain leukemia- and lymphoma-derived cells [6], [7] without similarly affecting their non-transformed counterparts. Further development and clinical trials led to the approval of the modified boronic dipeptide Pyz-Phe-boroLeu, known as Bortezomib or Velcade®, as a drug for the treatment of multiple myeloma [8], [9], [10], [11]. Most synthetic proteasome inhibitors are short peptides that mimic protein substrates. Typically, the pharmacophore that reacts with and inhibits the threonine residue in the 20S proteasome's active site is bound to the carboxyl residue of the peptide [12]. Some of the typical synthetic inhibitors are peptide aldehydes, peptide vinyl sulfones, peptide boronates, and peptide epoxyketones [for review, see [13]]. Most notable among the natural, bacterially derived non-peptide inhibitors is *claso*-lactacystin-β-lactone (Omuralide) [14]. Related drugs such as Salinosporamide A (NPI-0052) and Carfilzomib (PR-171) are currently in advanced clinical trials [15], [16], [17]. However, despite the extensive efforts invested in proteasome inhibitor development, there is a growing need for novel inhibitory molecules, due to the emergence of drug-resistant cells and the variable effects of existing inhibitors on different cells.

Most of the current assays for proteasome inhibition are based on cell-free assays [18], [19], which require purification of 26S or 20S proteasomes from different sources. Such assays may, in principle, be adapted to high-throughput screens, yet they may fail to predict the inhibitory activity in live cells. To overcome this problem, cell-based screens have been incorporated into the drug discovery process. For example, a modified “classical” method for measurement of the chymotrypsin-like, trypsin-like, or caspase-like proteasome activities in cultured cells [20] is currently available from Promega Corporation. A number of fluorescent reporter molecules have been also usefully employed to monitor the activity of the proteasome. Dantuma et al constructed a fusion of GFP to Ubiquitin (Ubi[G76V]-GFP) using a standard peptide bond at the N-terminus [21], Another proteasome sensor construct, which is a GFP fusion to an artificial peptide, CL1, identified in yeast has been designed by Bence et al ([22]. The Andreatta group and BD Biosciences Clontech has introduced a sensor cell line expressing a GFP fusion protein with a fragment of the mouse ornithine decarboxylase (MODC), which is degraded by the proteasome without the requirement for ubiquitination [23]. An additional reporter cell line, based on the stable expression of a p27^kip1^-GFP fusion was recently employd for the discovery of a novel proteasome inhibitor, argyrin A [24]. The common feature of most of these GFP-fused reporters is that they are based on proteins rapidly degraded by the proteasome under normal conditions, leading to very low fluorescence of the cells, while following inhibition of proteasome activity, the overall fluorescent signal of the cells rapidly increases as a result of accumulation of the reporter proteins.

In this article, we describe a novel, image-based screening approach, which utilizes an H1299 reporter cell line, stably expressing a fluorescent “Proteasome Inhibition Reporter” (PIR) protein. The screen is based on the finding that upon inhibition of proteasomal activity, this fluorescent reporter molecule translocates from the cytoplasm to the nucleus. Our findings demonstrate that this approach is highly sensitive, and compatible with high throughput microscopy. In the screen reported herein, we tested the Diversity Set of the NIH/NCI chemical library, and identified four compounds with pronounced proteasome inhibitory activity, three of which are novel inhibitors.

## Materials and Methods

### DNA Constructs, Generation of a Stable Reporter Cell Line and Transient Transfection

To construct a YFP-tagged Proteasome Inhibitor Reporter (PIR) protein, cDNA encoding a full-length human p53 R175H mutant was amplified by PCR from a cDNA clone, and three consecutive lysine residues in the bipartite Nuclear Localization Signal (NLS) were replaced with alanines by PCR-based, site-directed mutagenesis [25] [26]. The DNA fragment was cloned into the BglII and NotI sites of pLPCX retroviral vector (Clontech) in-frame to the N-terminus of the yellow fluorescent protein (YFP), using the NotI/ClaI restriction sites. The PIR protein was expressed in an H1299 non-small cell lung carcinoma cell line, following retroviral infection, and the cells were cultured in RPMI 1640 medium containing 10% fetal bovine serum, 2 mM glutamine and 1% penicillin-streptomycin (all from Sigma-Aldrich) in a humidified atmosphere of 5% CO_2_ at 37°C. Fluorescent cells were isolated by flow cytometry, and single-cell cloning was used to generate a morphologically uniform cell population.

Plasmids for the expression of human MDM2 were kindly provided by B. Vogelstein (wild type MDM2), A. Levine [MDM2 Δ9-58] and A. Ciechanover [MDM2 Ser 440]. Transfections employing plasmid DNA were carried out using Lipofectamine 2000™ reagent (Invitrogen) as per the manufacturer's instructions. For RNA interference, PIR cells were transfected with 50 pM control or MDM2 siRNA oligonucleotides (Dharmacon, ON-TARGET*plus* SMARTpool), with Dharmafect 2 (Dharmacon) according to the manufacturer's protocol.

### Immunofluorescence Microscopy

Cells were cultured on glass coverslips, fixed, and permeabilized for 2 min in phosphate-buffered saline (PBS) containing 0.5% Triton X-100 and 3% formaldehyde, and post-fixed with 3% formaldehyde in PBS for 30 min. The cells were then rinsed and stained with polyclonal anti-β-catenin antibody (Sigma) or a mixture of anti-MDM2 monoclonal antibodies SMP14, 2A10, and 4B11 for 1 h (hybridoma cells were kindly provided by A. Levine), washed, and further incubated with Cy3-conjugated goat anti-mouse IgG (Enco). Images were acquired using the DeltaVision system (Applied Precision Inc.).

### Compound Library

The chemical compound library screened here for proteasomal inhibitors consisted of the NCI Diversity Set, containing 1,992 low molecular weight synthetic compounds selected from and representing nearly 140,000 compounds available from the NCI DTP chemical library (http://dtp.nci.nih.gov/branches/dscb/diversity_explanation.html).

The library compounds were dissolved in dimethyl sulfoxide (DMSO) to a concentration of 10 mM, placed in 96-well plates, and stored at −70°C for future use.

### Image-Based Screening Assay for Proteasome Activity

PIR-expressing H1299 cells were seeded at a density of 800 cells per well in 384-well assay plates (F-bottom, µClear, black, tissue-culture-treated) (Greiner Bio-One). Cells were cultured for 24 h and treated with the library compounds at two concentrations (1 and 10 µM) in RPMI 1640. In each assay plate, cells in 24 wells treated with 0.2% DMSO alone were used as controls. As a positive control, 1 µM MG132 was added to a single column of the assay plate. Following 12 hours of incubation, cells were fixed in 3% paraformaldehyde for 20 min, then washed with PBS and screened for localization of the PIR protein.

### Microscope Automation and Image Processing

WiScan™ Cell Imaging System (Idea Bio-Medical) was used for this screen [27]. The system is based on an IX71 microscope (Olympus), equipped with a fast laser AutoFocus device [28] and an automated stage. Thirty-six fields were acquired from every well using a 60x/0.9 air objective, stored, and tiled into montages to detect consistent effects. Scoring of the nuclear translocation of the fluorescent PIR protein was carried out manually.

### In Vitro Proteasome Activity Assay

For measuring proteasome activity, purified 20S or 26S proteasomes prepared from rabbit muscles were incubated at a final concentration of 16 nM, with 100 µM fluorogenic 7-amido-4-methylcoumarin (AMC) tetrapeptide substrate Suc-LLVY-AMC (Bachem) and the stated concentration of the hit compounds in the presence of 100 µl of assay buffer (50 mM Hepes, 5 mM MgCl_2_, 2 mM ATP and 1 mM DTT). The well-documented proteasomal inhibitor MG132 was used as a positive control, and an equivalent volume of solvent as a negative control. The time-dependent increase of hydrolyzed AMC groups was measured in a 96-well plate equilibrated to 37°C, using a Varioscan multi-well plate reader (Thermo Fisher Scientific, Inc.) in a kinetic mode, in which the recording intervals were set to 1 minute. The excitation wavelength was 370 nm; fluorescence emission was recorded at 465 nm.

### Immunoblotting

H1299-PIR cells were lysed with radioimmune precipitation assay buffer (1% NP-40, 1% sodium deoxycholate, 0.1% SDS, 150 mM NaCl, 50 mM Tris, pH 8.0) containing a protease inhibitor cocktail from Roche Applied Science. Protein extracts were subjected to 8% SDS-PAGE, transferred to a nitrocellulose membrane (Whatman), and probed with monoclonal anti-p53 (DO1, Santa Cruz Biotechnology), anti-ubiquitin (Covance), anti-β-catenin (Sigma) and anti-β-tubulin (Sigma) primary antibodies.

For sub-cellular fractionation, cells were harvested and resuspended in ice-cold hypotonic lysis buffer [10 mM HEPES pH 7.9, 1.5 mM MgCl2, 10 mM KCl, 1 mM DTT, supplemented with a complete protease inhibitor cocktail (Roche)], incubated on ice for 15 min, then NP40 was added, to a final concentration of 0.6%. The samples were vortexed for 10 s and immediately centrifuged at 12,000 g for 30 s. The supernatant (cytoplasmic fraction) was transferred to a fresh tube. The nuclei pellet was washed once with hypotonic lysis buffer, and lysed with SDS sample buffer (100 mM Tris–HCl pH 6.8, 2% SDS, 100 mm DTT, and 10% glycerol).

### Cell Viability Assay

The effect of each hit compound on cell viability was tested at 11 different concentrations, ranging from 0.1–100 µM. PIR cells were plated onto 384-well microplates for 24 hours, and then treated for 48 h with the library compounds. Cell viability was determined using the colorimetric AlamarBlue® (Invitrogen) viability assay, according to the manufacturer's instructions. Results are expressed as GI_50_; namely, the compound concentration that reduces the AlamarBlue® score by 50%, compared to untreated controls.

## Results

### Development of an H1299-PIR Reporter Cell Line and Its Application for an Image-Based, High-Throughput Screening for Proteasome Inhibitors

**Figure 1:**
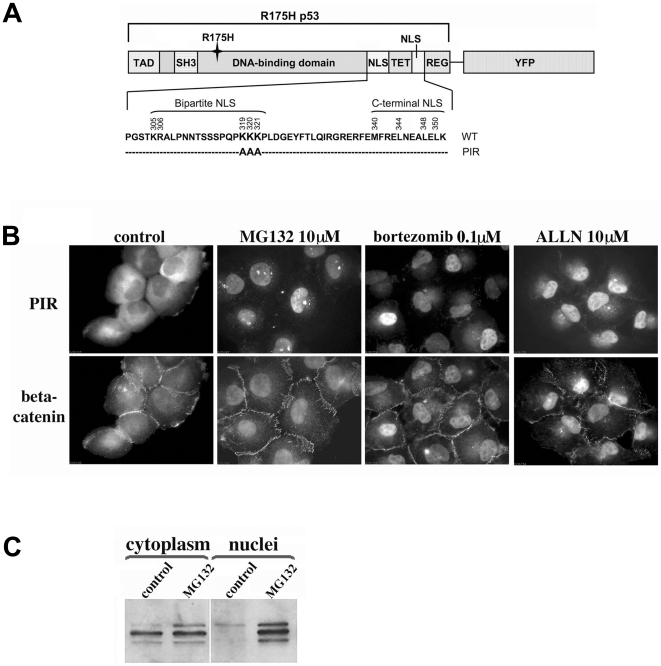
Engineering and validation of the PIR reporter system. (**A**) Schematic representation of the PIR protein. PIR consists of yellow fluorescent protein (YFP) fused to the C-terminus of the human p53 mutant R175H, carrying a triple mutation in the bipartite NLS in which three consecutive lysine residues were replaced with alanines K319A, K320A, and K321A. (**B**) Nuclear accumulation of the PIR protein upon treatment with proteasome inhibitors. PIR cells were exposed to MG132, Bortezomib, and ALLN at the indicated concentrations for 6 h. PIR-associated YFP-fluorescence (upper panel) and immunostaining with anti-β-catenin antibody (lower panel) are seen. (C) PIR cells were incubated for 6 h without proteasome inhibitor (control), or with 10 µM MG132. Fractionated cytoplasm and nuclear lysates were analyzed by Western blot with anti-p53 antibody. A band corresponding to PIR was detected in the nuclear fraction only following treatment with MG132.

The PIR protein, used here for proteasome inhibitor screening, was constructed of yellow fluorescent protein (YFP) fused to the C-terminus of the human p53 R175H mutant. The cytoplasm-to-nucleus transport of this p53 mutant was attenuated by additional triple mutation in the bipartite NLS in which three consecutive lysine residues were replaced with alanines (K319A, K320A, and K321A), as described by O'Keefe et al [29]. In agreement with previous reports, under normal cell culture conditions, this point mutation leads to cytoplasmic localization of the PIR protein in H1299 cells [29], [30], confirming that the three basic residues at position 319–321 are indeed part of a nuclear localization signal. However, upon treatment with known proteasome inhibitors (e.g. MG132, Bortezomib and ALLN), PIR translocates into the nucleus in a manner reminiscent of p53 mutated in its NLS (Fig. 1B upper panel) [31]. β-catenin, whose cellular levels are primarily regulated by the proteasome, underwent similar nuclear translocation in response to proteasome inhibition (Fig. 1B, lower panel), yet this was accompanied by significant stabilization and increase in its quantity [32] unlike PIR, whose overall levels did not markedly change. To validate these results, we quantified the levels of PIR in the nuclear and cytoplasmic fractions of treated and control H1299-PIR cells using immunoblotting. This assay confirmed our microscopy-based observation, and pointed to a ∼3-fold increase in the nuclear/cytoplasmic ratio of PIR, in response to MG132 treatment (Fig. 1C).

To assess the sensitivity of the PIR cell-based assay, H1299-PIR cells were incubated for 8 hours with different concentrations (0.01–10 µM) of known proteasome inhibitors (MG132 and Bortezomib). The cells were then fixed and scored for nuclear translocation of PIR. The score (EC_50_) refers to the concentration of inhibitor needed to induce nuclear translocation of PIR in 50% of the treated cells. This test indicated that in our assay, the EC_50_ values for MG132 and Bortezomib were 0.5 µM, and 0.05 µM, respectively. These values favorably compare with those reported for other detection systems, such as the commercial Living Colors HEK 293 ZsGreen Proteasome Sensor system (Clontech), which detects MG132 at 2.5 µM (after 20 hours of treatment using flow cytometry) [23], or for the Ubi[G76V]-GFP-based reporter system (BioImage), in which the reported EC_50_ value for MG-132 was approximately 1.0 µM [21]. Thus, H1299-PIR cells appear to be sensitive reporters, capable of detecting the activity of proteasome inhibitors in a cell-based assay.

### Nuclear Accumulation of Endogenous MDM2 in Response to Proteasome Inhibition Is Responsible for PIR Nuclear Translocation

**Figure 2:**
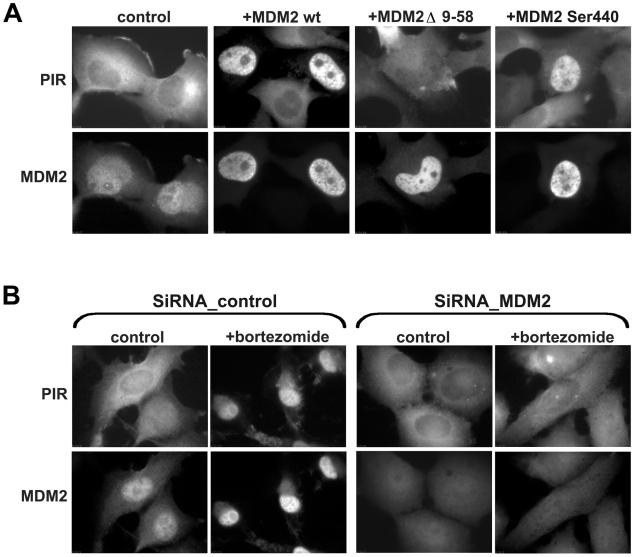
MDM2 promotes PIR nuclear translocation. (A) Overexpression of MDM2 results on the PIR nuclear localization without additional stimuli. PIR cells were transfected with wild-type MDM2, MDM2 mutant deficient on p53 binding (Δ 9-58), or MDM2 mutant with abolished E3 ligase site (Ser 440). Cells expressing both p53 and MDM2 were visualized by immunofuorescence staining with the anti-MDM2 monoclonal antibodies. PIR has a nuclear localization in the cells expressing wt MDM2 and MDM2 (Ser 440), and remains cytoplasmic in the cells transfected with MDM2 (Δ 9-58). (B) Mdm2 siRNA prevents bortezomide–induced translocation of PIR to the nucleus. PIR cells were transiently transfected with 200 pmol control-siRNA or Mdm2-siRNA. Forty-eight hours after transfection, bortezomide (0.1 µM) was added for an additional 6 h, and immunofuorescence staining for MDM2 was performed as described in Materials and Methods.

To further characterize the mode of PIR nuclear translocation upon proteasome inhibition, we have considered the possibility that proteasome-sensitive p53 binding proteins, are responsible for carrying PIR into the nucleus. Towards this end, MDM2, a p53 E3 ubiquitin ligase and a known target of proteasome-dependent degradation, was transfected into PIR cells, and its localization was assessed by immunofuorescence microscopy. As expected, endogenous MDM2 labeling in the PIR-H1299 cells was relatively faint and mostly nuclear while PIR was mainly localized to the cytoplasm (Figure 2a). In contrast, in the cells transfected with wild type MDM2, both the fluorescent PIR and MDM2 translocated to the nucleus. This suggests that MDM2 can transport NLS-deficient PIR from the cytoplasm into the nucleus, perhaps via the NLS of MDM2, consistently with previous studies suggesting that MDM2 can promote the nuclear import of ΔNLS p53 [31]. Interestingly, PIR remained cytoplasmic in cells over-expressing a mutated MDM2 lacking the p53 binding site (MDM2 Δ 9-58), suggesting that the interaction between the two proteins is needed for their cotranslocation to the nucleus. On other hand, MDM2 mutant with point mutation that abolishes its E3 ubiquitin ligase function (MDM2 Ser440) induced PIR nuclear localization similar to the wild type molecule.

To check whether MDM2 expression is critical for PIR nuclear translocation, we performed siRNA-mediated knockdown of MDM2 expression in PIR-cells, and then treated the cells with proteasome inhibitors (Figure 2b). It was found that when MDM2 levels in the knocked-down cells were reduced, PIR remained cytoplasmic even following treatment with proteasome inhibitors, indicating that MDM2 is an essential player in the nuclear localization of NLS-deficient PIR.

### Screening for Novel Proteasome Inhibitors in the Diversity Set of the NIH/NCI Chemical Library

**Figure 3:**
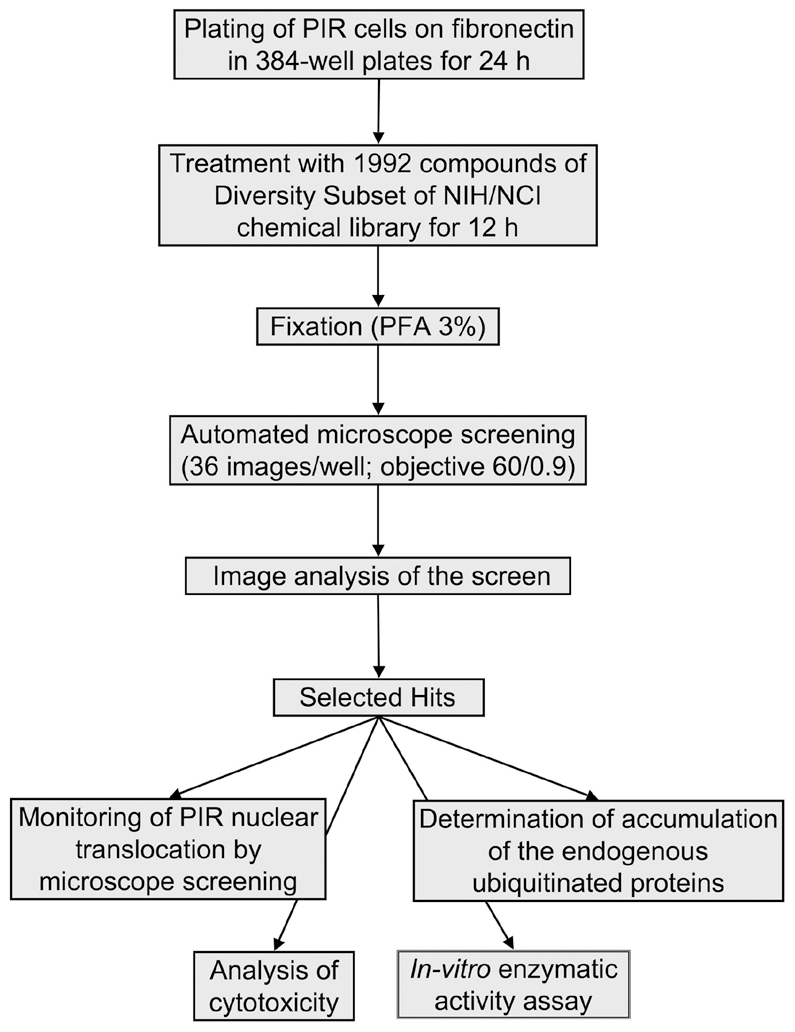
A flow chart of the screening procedure. For the screening assay, H1299-PIR reporter cells were plated in 384-well plates for 24 h and treated with compounds of the NCI Diversity Set library at two concentrations (1 and 10 µM), one compound per well. Following 12 hours of incubation, cells were fixed in 3% paraformaldehyde and screened for PIR cellular localization with WiScan™ automated microscope system. Acquired images were analyzed for PIR nuclear translocation, and selected hits were confirmed by microscopy-based and biochemical methods, and tested for compound cytotoxicity.

To assess the potential use of the cytoplasm-to-nucleus translocation of PIR in high-throughput, microscopy-based screening for novel proteasome inhibitors, we tested 1,992 low molecular weight compounds comprising the NCI Diversity Set chemical library. A flow chart depicting the screening procedure is shown in Figure 3, and described in the Experimental Procedures. Following the initial automated screen, the images of the affected cells were inspected manually and a secondary screen was performed, in which hit compounds were tested at multiple concentrations, and directly compared to the well-established proteasome inhibitor MG132. This procedure resulted in the discovery of four compounds that induced nuclear translocation of PIR, indicating a hit rate of ∼0.2%. As summarized in Table 1, all four compounds detected in the primary screen were confirmed by manual inspection.

**Table 1:** The hit compounds identified in the PIR screen as potent proteasomal inhibitors.

	NSC3907	NSC99671	NSC310551	NSC321206
**Chemical name**	8-Hydroxyquinoline salicylate	2-[2-[(1,3-dimethyl-2-oxo-6-sulfanylidene-7H-purin-8-yl)sulfanyl] ethyl]isoindole-1,3-dione	copper; [(6-methylpyridin-2-yl)methylideneamino]-[methylsulfanyl (sulfoniu mylidene]methyl) azanide	bromocopper; (dipyridin-2-ylmethylideneamino)-[methylsulfanyl(sulfoniumylidene) methyl]azanide
**Molecular formula**	C16H13NO4	C17H15N5O3S2	C18H22CuN6S4	C13H12BrCuN4S2
**EC50**	5 mM	15 mM	0.25 mM	0.1 mM

### Biochemical Validation of the Inhibitory Effects of the Hit Compounds

**Figure 4:**
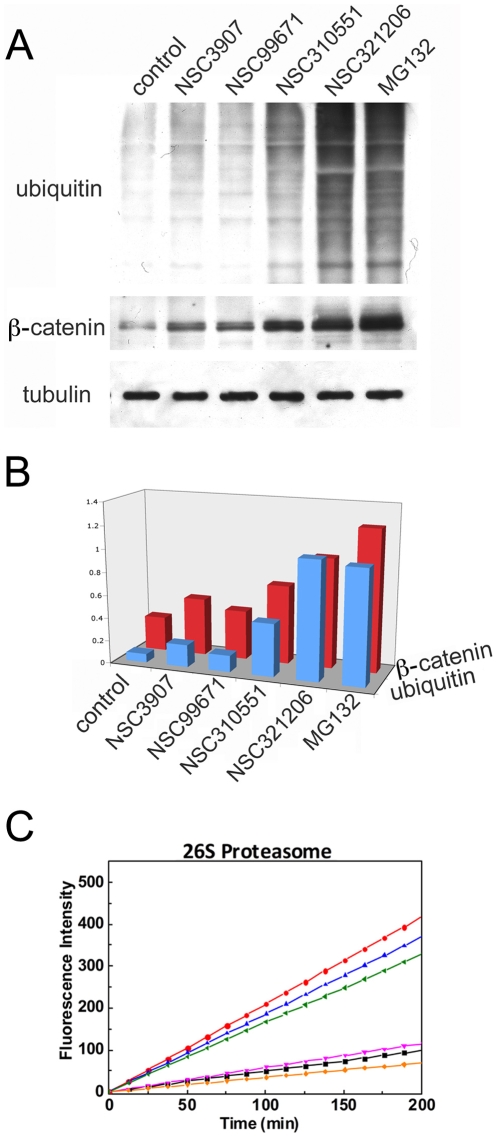
Positive hit compounds inhibit 26S proteasome. (**A**) PIR cells were treated with hit compounds and MG132 for 6 hr at the following concentrations: NSC3907 – 20 µM; NSC99671 – 50 µM; NSC310551 – 0.3 µM; NSC321206 – 0.15 µM; and MG132 - 5 µM). Whole cell lysates were immunoblotted for ubiquitin (upper panel) and β-catenin (middle panel). Tubulin (lower panel) was used as an internal loading control. (**B**) Quantitation of the amounts of ubiquitinated proteins (blue) and β-catenin (red) in the absence of and upon treatment with the different inhibitors. (**C**) Positive hit compounds inhibit purified proteasome *in vitro*. Purified 26S proteasomes from rabbit muscle were incubated for the indicated times in the presence of 30 µM of our positive hits (100 µM for NSC3907). MG-132 at a 5 µM concentration served as a positive control. NSC3907 (blue), NSC99671 (green), NSC310551 (magenta), NSC321206 (orange), without treatment (red) and MG-132 (black).

One characteristic outcome of proteasome inhibition is the accumulation of ubiquitinated proteins in the treated cells. To monitor the levels of ubiquitinated proteins that accumulated upon incubation with the novel inhibitors detected in our screen, H1299-PIR cells were treated with each of the inhibitors for 6 hours, at doses comparable to those that were used in the screen. Following incubation, cell extracts were analyzed by Western blot, using anti-ubiquitin and anti-β-catenin antibodies. As shown in Fig. 4A&B, accumulation of endogenous polyubiquitinated proteins, as well as elevated levels of β-catenin (a known target of the proteasome), at varying degrees, were caused by all four inhibitors, confirming their inhibitory effect on proteasomal degradation. The hit compounds NSC321206 (at a concentration of 0.15 µM) and NSC310551 (0.3 µM) were the most effective, demonstrating inhibitory activity comparable to that of 5 µM MG132. NSC99671 and NSC3907 (50 µM and 20 µM, respectively) displayed less of an inhibitory effect. It is noteworthy that the same concentrations that induced nuclear transport in the PIR assay, also resulted in accumulation of polyubiquitinated proteins and stabilization of β-catenin. Moreover, the potency of proteasomal inhibition, judged by these criteria, coincides nicely with the magnitude of the nuclear fluorescence signal detected in the PIR cell-based assay, upon inhibition with the different hit compounds.

To directly test the capacity of the four compounds to inhibit activity in mammalian proteasomes, we performed an *in vitro* activity assay in which the hit compounds were tested for their effects on the degradation of the model fluorogenic tetrapeptide LLVY-AMC by purified rabbit 26S proteasomes. As seen in Fig. 4C, all four compounds inhibited proteasomal degradation to varying degrees. Both NSC310551 and NSC321206 showed levels of inhibition comparable to that of MG132, with NSC321206 being the most effective inhibitor. NSC99671 displayed a moderate inhibitory effect, and NSC3907 had only a minor effect. The low potency of NSC3907 in inhibiting the purified proteasome was consistent with previous findings [33], [34], showing that this molecule (8-Quinolinol salicylate) can specifically inhibit the chymotryptic activity of the proteasome only in complex with intracellular copper. The fact that this compound was still picked up by our screen reflects an advantage of this cell-based assay.

### Effect of the Novel Proteasome Inhibitors on Cell Viability

**Figure 5:**
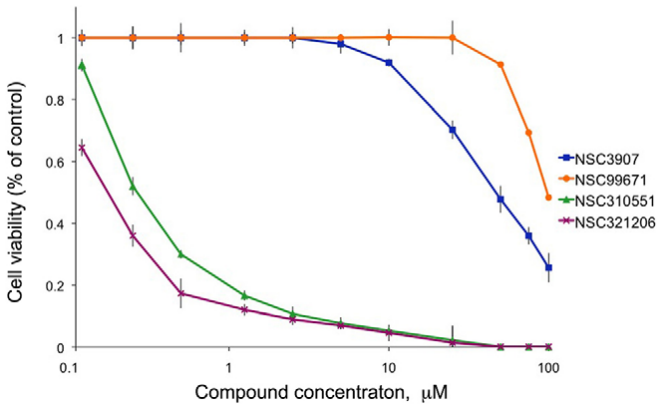
Effect of the hit compounds on the viability of PIR cells. PIR cells were treated with the active compounds for 48 hr at 11 concentrations ranging from 0.1 to 100 µM and the cells' viability was assessed by AlamarBlue assay. Results are expressed as the viability ratio of treated to untreated cells and represent the mean ± SD of 6 repeats.

Proteasome inhibitors are known to be particularly cytotoxic to malignant cells via multiple mechanisms [7], [35]. To directly test the effects of the new proteasome inhibitors discovered in this study on cell viability, we treated PIR-expressing H1299 cells for 48 h with each of the four compounds, at a wide range of concentrations, ranging from 0.1 to 100 µM. The cells were then subjected to an Alamar Blue viability assay, which quantifies the number of metabolically active cells. As shown in Fig. 5, all four compounds affect cell viability, or inhibit the growth of PIR-H1299 cells (independent of the presence of PIR), at different concentrations. NSC3907 and NSC99671 exhibited a relatively weak growth inhibition effect, with GI_50_ values of 47 µM and 96 µM, respectively, while NSC310551 and NSC321206 displayed a considerably stronger effect, with GI_50_ values of 0.27 µM and 0.17 µM, respectively. For all proteasome inhibitors examined in this screen, there was a high correlation between proteasomal inhibitory activity and cell viability.

In view of previous reports, indicating that malignant cells are significantly more sensitive to proteasome inhibition than their normal counterparts [7], [36], we compared the effect of the most effective inhibitory compound, NSC321206, toward normal breast epithelial cell line (MCF10A) and malignant breast carcinoma cells (MDM-MB-231) cell lines. As shown in Fig. S1, NSC321206 effectively eliminated all MDA-MB-231 cells at a concentration of 1 µM (GI_50_ value of 0.4 µM), while the non-malignant breast epithelial cell line (MCF10A) were only partially affected, at a considerably higher concentration of this compound.

To gain insights into the effects of our hit compounds on a wide variety of cells, we explored the published information on the effects of these compounds on the NCI-60 panel of human tumor cell lines used in the NCI Developmental Therapeutics Program (DTP) (http://dtp.nci.nih.gov). As seen in Figure S2, the four hit compounds showed cytotoxic effects (log_10_ GI_50_ <−4.0) against a variety of cell lines, whereas NSC321206 and NSC310551 demonstrated high cytotoxicity *in vitro* against all tested human cancer cell lines in the panel, with average negative log_10_ GI_50_ values of 7.2 and 6.6, respectively. The activity of NSC3907 was much lower, with a mean overall – log_10_ GI_50_ value of 5.3. NSC99671 was non- toxic for most of the lines (overall –log10 GI_50_ of 4.1). The most sensitive cell lines for all hit compounds were the leukemia cells, with overall -log_10_ GI_50_ equaling 7.73 for NSC321206, 7.028 for NSC310551, 6.249 for NSC3907, and 4.473 for NSC9967. These initial findings corroborate our *in vitro* results, and directly demonstrate the use of our novel proteasomal inhibitors as potential therapeutic agents in cancer.

## Discussion

Presently, few approaches for the high-throughput discovery of proteasome inhibitors exist [18], [19], [20], those that do, are mostly based on the use of biochemical techniques. Cell-based/image-based assays enables evaluation of potential proteasomal inhibitors that may not be detected using purified proteasomes and have several other advantages such as demonstrating that active compounds are cell-permeable and are sensitive to effects at multiple targets and nodes within a given pathway, as opposed to a strict cell-free assay that focuses on one particular target, such as degradation of a particular substrate by a purified proteasome. The main motivation directing the development of PIR was to establish a method enabling the assessment of proteasomal inhibition in a cellular context, based on the unequivocal translocation of a fluorescent reporter protein from the cytoplasm to the nucleus upon proteasomal inhibition, without grossly affecting its overall levels. We found this approach to be highly specific, with essentially no false positives, in contrast to existing cell-based screens for proteasomal inhibitors that monitor the accumulation of fluorescent signals from direct proteasomal substrates [21], [22], [23], that appear to be sensitive to autofluorescence and to the fluorescence-quenching effects of the screening molecules, as well as to variations in cell geometry, some of which may be induced, directly or indirectly, by proteasomal inhibition.

The design of the PIR reporter protein is based on a p53 R175H mutant which, in contrast to the short-lived, WT p53, has a significantly longer half-life (several hours), presumably due to its reduced susceptibility to proteasomal degradation [37]. As a result, the overall concentration of PIR in cells is only marginally affected by treatment with proteasomal inhibitors such as MG132 (Fig. 1C). In the PIR assay, monitoring proteasomal inhibition is based on intracellular translocation of the reporter protein from the cytoplasm to the nucleus, in response to proteasomal inhibition. It is noteworthy that PIR was found to be particularly suitable for high throughput screening for proteasomal inhibitors, due to the unambiguous quantification of nuclear vs. cytoplasmic fluorescence.

Several proteins such as p53, MDM2 and β-catenin were previously reported to accumulate in the nucleus following treatment with proteasome inhibitors [32], [38], [39], [40], [41], [42]. Beyond the fact that these are all target substrates of the ubiquitin-proteasome pathway, their translocation to the nucleus is mediated via recognition of their NLS by cytosolic nuclear transport receptors [apart from β-catenin, which is imported into the nucleus by direct binding to the nuclear pore machinery [43]]. Typically, deletion or mutation of the NLS disrupts their nuclear import. Under normal cell culture conditions, PIR, which is based on R175H p53 with a mutated bipartite NLS [29], loses its ability to translocate to the nucleus and, therefore, accumulates in the cytoplasm. Even treatment with etoposide, which is a strong inducer of p53 cytoplasm-to-nucleus transport, fails to promote this translocation. Our studies provide some clues as to the mechanism underlying PIR translocation to the nucleus upon proteasomal inhibition. Specifically, we propose a model suggesting that nuclear entry of this molecule is driven by yet another protein, which is highly sensitive to proteasomal degradation, contains a nuclear localization domain, and is capable of piggy-backing PIR into the nucleus. Furtheremore, we provide evidence suggesting that this nuclear translocator of PIR is, in fact, the p53 E3 ubiquitin ligase MDM2, which was shown to translocate into the nucleus upon proteasome inhibition (Xirodimas et al 2001). MDM2 promotes PIR nuclear import in transiently transfected cells over-expressing wild type MDM2, in the absence of additional stimuli (Fig. 2). Upon proteasome inhibition, the levels of MDM2 dramatically increase and, in turn, drive PIR into the nucleus. We also demonstrated that the ability of MDM2 to bind p53 is critical for such translocation, unlike its E3 ubiquitin ligase function. Further compelling support for our model comes from the fact that the translocation of the PIR protein to the nucleus, induced by proteasome inhibition, is abolished by MDM2 knockdown, confirming that the interaction between the two proteins results in colocalization in the nucleus. This is consistent with previous reports showing that wild type p53 (as well as its homologue p73) and MDM2 may facilitate the shuttling of each other from the cytoplasm into the nucleus [31], [44] and vice versa.

In conclusion, the novel cell-based screen described here appears to be a robust and highly sensitive tool for the identification on new proteasome inhibitors. It is based on the stabilized MDM2-dependent accumulation of the PIR molecule in the nucleus, and is compatible with microscopy-based high throughput screening technology. Further characterization of the new inhibitors discovered using this approach, as well as the development of a combined ultra high resolution / high content screen, based on the PIR cells, are currently underway.

## Supporting Information

Figure S1 Differential effect of NSC321206 on human malignant breast cells (MDM-MB-231) and human non-malignant breast epithelial cells (MCF10A). The two cell lines were treated with NSC321206 for 48 hr at 6 concentrations ranging from 0.025 to 2.5 µM and the viability was assessed using the AlamarBlue assay. Results are expressed as the viability ratio of treated to untreated cells and represent the mean ± SD values of 6 repeats.
(2.12 MB TIF)

Figure S2 In vitro cytotoxicity of the hit compounds on an NCI-60 panel of human tumor cell lines. Results are based on data from anti-cancer drug screening against the full panel of 60 human cancer cell lines, conducted as part of the Developmental Therapeutics Program at the National Cancer Institute (http://dtp.nci.nih.gov). The panel is divided into nine sub-panels representing diverse cancer cell types, including leukemia, melanoma, and cancers of the lung, colon, kidney, ovary, breast, prostate, and central nervous system. Results obtained with this test are expressed as the -log of the molar concentration that inhibited cell growth by 50% (-log GI50 >4.00 for active compounds).
(4.24 MB TIF)
