# Supplementary figures and images for: Discovery of Novel Proteasome Inhibitors Using a High-Content Cell-Based Screening System
Source: PLoS One. 2009 Dec 30;4(12):e8503. doi: 10.1371/journal.pone.0008503 (PMC2797363; doi:10.1371/journal.pone.0008503)

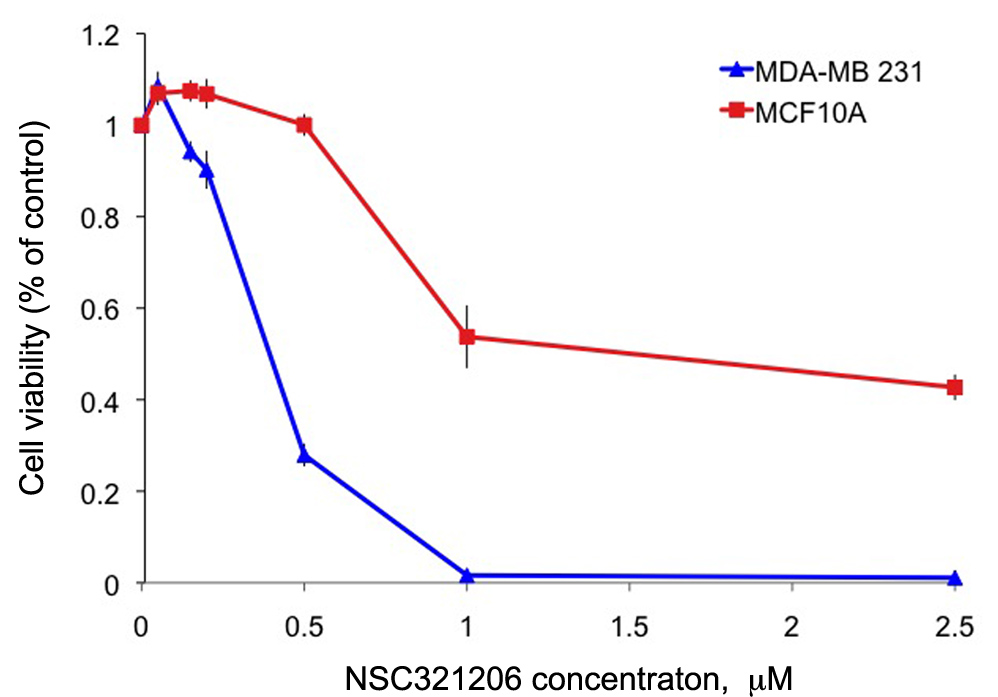

Supplement: Figure S1 — Differential effect of NSC321206 on human malignant breast cells (MDM-MB-231) and human non-malignant breast epithelial cells (MCF10A). The two cell lines were treated with NSC321206 for 48 hr at 6 concentrations ranging from 0.025 to 2.5 µM and the viability was assessed using the AlamarBlue assay. Results are expressed as the viability ratio of treated to untreated cells and represent the mean ± SD values of 6 repeats. (2.12 MB TIF) [file pone.0008503.s001.tif]

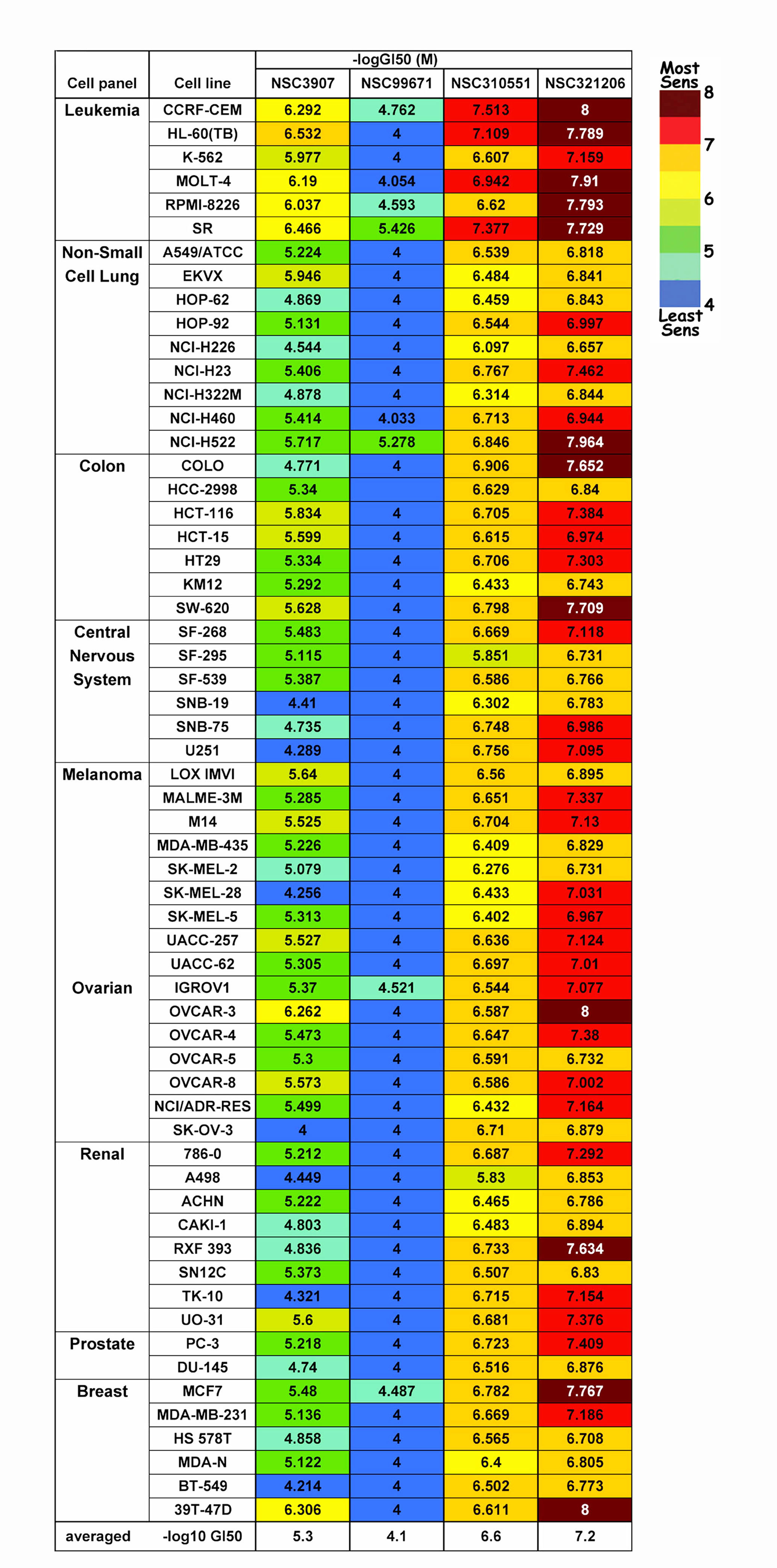

Supplement: Figure S2 — In vitro cytotoxicity of the hit compounds on an NCI-60 panel of human tumor cell lines. Results are based on data from anti-cancer drug screening against the full panel of 60 human cancer cell lines, conducted as part of the Developmental Therapeutics Program at the National Cancer Institute (http://dtp.nci.nih.gov). The panel is divided into nine sub-panels representing diverse cancer cell types, including leukemia, melanoma, and cancers of the lung, colon, kidney, ovary, breast, prostate, and central nervous system. Results obtained with this test are expressed as the -log of the molar concentration that inhibited cell growth by 50% (-log GI50 >4.00 for active compounds). (4.24 MB TIF) [file pone.0008503.s002.tif]
